# HIV Drug Resistance and Its Impact on Antiretroviral Therapy in Chinese HIV-Infected Patients

**DOI:** 10.1371/journal.pone.0054917

**Published:** 2013-02-06

**Authors:** Hui Xing, Yuhua Ruan, Jingyun Li, Hong Shang, Ping Zhong, Xia Wang, Lingjie Liao, Hanping Li, Min Zhang, Yile Xue, Zhe Wang, Bin Su, Wei Liu, Yonghui Dong, Yanling Ma, Huiqin Li, Guangming Qin, Lin Chen, Xiaohong Pan, Xi Chen, Guoping Peng, Jihua Fu, Ray Y. Chen, Laiyi Kang, Yiming Shao, The Chinese National HIVDR Surveillance and Monitoring Network

**Affiliations:** 1 State Key Laboratory for Infectious Disease Prevention and Control, and National Center for AIDS/STD Control and Prevention, Chinese Center for Disease Control and Prevention, Beijing, China; 2 AIDS Research Department, Institute of Microbiology and Epidemiology Academy of Military Medical Science, Beijing, China; 3 Key Laboratory of Immunology of AIDS, Ministry of Health, the First Affiliated Hospital, China Medical University, Shenyang, China; 4 Department of AIDS and STD, Shanghai Municipal Center for Disease Control and Prevention, Shanghai, China; 5 Henan Center for Disease Control and Prevention, Zhengzhou, China; 6 Anhui Center for Disease Control and Prevention, Hefei, China; 7 Guangxi Center for Disease Control and Prevention, Nanning, China; 8 Xinjiang Center for Disease Control and Prevention, Urumqi, China; 9 Yunnan Center for Disease Control and Prevention, Kunming, China; 10 Yunnan Infectious Disease Hospital, Kunming, China; 11 Sichuan Center for Disease Control and Prevention, Chendu, China; 12 Shenzhen Center for Disease Control and Prevention, Shenzhen, China; 13 Zhejiang Center for Disease Control and Prevention, Hangzhou, China; 14 Hunan Center for Disease Control and Prevention, Changsha, China; 15 Hubei Center for Disease Control and Prevention, Wuhan, China; 16 Shandong Center for Disease Control and Prevention, Jinan, China; 17 National Institute of Allergy and Infectious Diseases, U.S. National Institutes of Health, U.S. Embassy Beijing, Beijing, China; National AIDS Research Institute, India

## Abstract

**Background:**

Highly active antiretroviral therapy (HAART) has significantly decreased mortality among Chinese HIV patients. However, emerging HIV drug resistance (HIVDR) poses a growing threat to the long-term success and durability of HAART.

**Methods:**

Three cross-sectional surveys were conducted across the country from 2004 to 2006, respectively. Patients completed a questionnaire and provided blood for CD4 cell count, HIV viral load (VL), and HIV resistance genotyping. Factors associated with HIVDR were identified by logistic regression.

**Results:**

3667 unique patients were included across the three surveys. Among 2826 treatment-experienced patients, median duration of treatment was 17.4 (IQR 8.6–28.4) months and HIVDR was identified in 543 (19.2%). Factors significantly associated with HIVDR included ART drug distribution location, CD4 cell count, initial HAART regimen, self-reported medication adherence, and province.

**Conclusions:**

Virologic failure increased over time on therapy but a significant proportion of patients in failure had no resistance mutations identified, suggesting that treatment adherence is suboptimal and must be emphasized. Due to the significantly higher risk of HIVDR in certain provinces, additional steps to reduce HIVDR should be taken.

## Introduction

The rapid expansion of highly active antiretroviral therapy (HAART) in resource-limited settings has markedly improved the prognosis of patients infected with HIV [1]. These benefits, however, can be compromised by the development of HIV drug resistance (HIVDR) [2], [3], making treatment less effective [4], [5]. Drug resistance is increasingly problematic in developing countries, which rely heavily on first-line generic drugs and have very limited second-line treatment options. Factors associated with the development of drug resistance include inadequate suppression of virus replication due to suboptimal treatment regimens, difficulty adhering to complex and toxic regimens, and initiation of therapy late in the course of HIV infection [6]. To understand better the prevalence and risk factors of HIVDR in China, three cross-sectional surveys were conducted over time by the Chinese National HIVDR Surveillance and Monitoring Network.

## Methods

### Study Design and Setting

The China National Free Antiretroviral Treatment Program [1], [2], [7], [8] was initiated in 2002 among former plasma donors who contracted the virus in the mid-1990s [9], then expanded beginning in 2003 to treat all HIV-infected patients across mainland China who met the national treatment guidelines of: (1) CD4 cell count <350/mm^3^ (increased from 200/mm^3^ in 2008); (2) total lymphocyte count <1,200/mm^3^; or (3) World Health Organization (WHO) stage III or IV disease [9], [10]. First-line HAART regimens consist of [azidothymidine (AZT) or stavudine (D4T)] + [didanosine (DDI) or lamivudine (3TC)] + [nevirapine (NVP) or efavirenz (EFV)]. AZT, D4T, DDI, and NVP are generically produced in China, whereas 3TC and EFV are branded drugs that became available in 2005. Second-line drugs, including tenofovir (TDF) and lopinavir/ritonovir (LPV/r), were introduced in limited fashion since 2008 but have not yet scaled up widely.

### Patient Recruitment Algorithms for Cross-sectional Surveys

**Figure 1:**
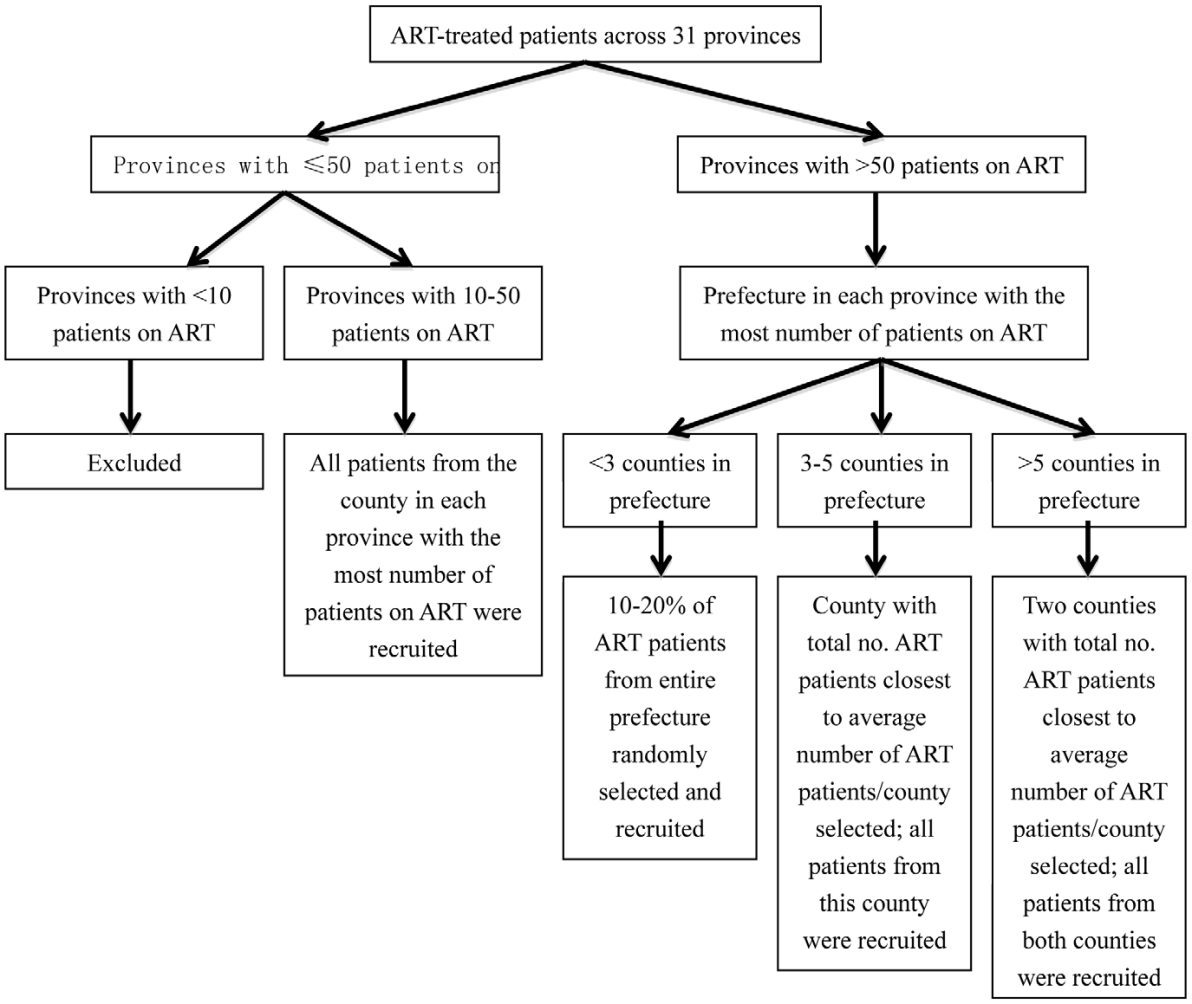
Patient recruitment algorithm for antiretroviral therapy experienced patients screened for the 2004, 2005, and 2006 cross-sectional surveys.

The Chinese National HIVDR Surveillance and Monitoring Network consists of 4 central laboratories (National Center for AIDS/STD Control and Prevention [NCAIDS], Shanghai Municipal Center for Disease Control and Prevention (CDC), Chinese Medical University Center for AIDS Research, and Institute of Microbiology and Epidemiology of the Chinese Academy of Military Medical Sciences) and laboratories from 30 provincial CDCs. Cross-sectional surveys were conducted nationally among HIV-infected adults ≥18 years in 2004, 2005, and 2006. For treatment-experienced patients, screening and recruitment into the study was performed per Figure 1. In 2006, in addition to this algorithm, all patients previously surveyed in 2004 or 2005 and could be located were also followed-up. County was the minimal sampling unit and sampling proportion was reverse with the number of treated patients in the county so that the strategy could result in a representative population. After informed consent, all patients completed a standardized questionnaire for demographics, HIV risk factors, and ART history. Treatment adherence was measured by self-reported missed doses in the previous month. A blood sample was collected for CD4 cell count and HIV viral load (VL). Genotypic resistance testing was performed if VL was ≥1000 copies/mL.

All subjects provided written informed consent to participate in this study. The institutional review board (IRB) of the NCAIDS, China CDC approved this study.

### Laboratory Analysis

CD4 cell count was measured by flow cytometry in provincial CDCs. The four state central laboratories performed the HIV VL and drug resistance tests. Plasma HIV-1 RNA was quantified with real-time nucleic acid sequence based amplification (NASBA; NucliSense Easy Q, bioMerieux, France) or Amplicor HIV-1 monitor test (COBAS®, Roche Applied Science, Germany). In samples with VL ≥1000 copies/ml, HIVDR genotyping was performed by in-house polymerase chain reaction (PCR) as previously described [11], [12]. The resulting fragment of the HIV-1 *pol* gene (protease, amino acids 1–99; and part of reverse transcriptase, amino acids 1–252) was analyzed for drug resistance mutations using the Stanford HIV Drug Resistance Database (http://hivdb.stanford.edu). We included mutation results that conferred low-, intermediate-, and high- level resistance [13].

### Statistical Analysis

For patients sampled more than once across the three surveys, data from the first survey showing drug resistance were included in this analysis. If no drug resistance was identified, data from the last survey were included. Data from the three surveys were combined. Demographic variables were described with descriptive statistics. Risk factors for developing HIVDR were analyzed by logistic regression. Variables associated with resistance in univariate analyses (*p*-value<0.05) and those clinically meaningful were included in the multivariable regression model. Tests were two-sided, with a *p*-value<0.05 indicating statistical significance.

## Results

### Demographic Characteristics

**Figure 2:**
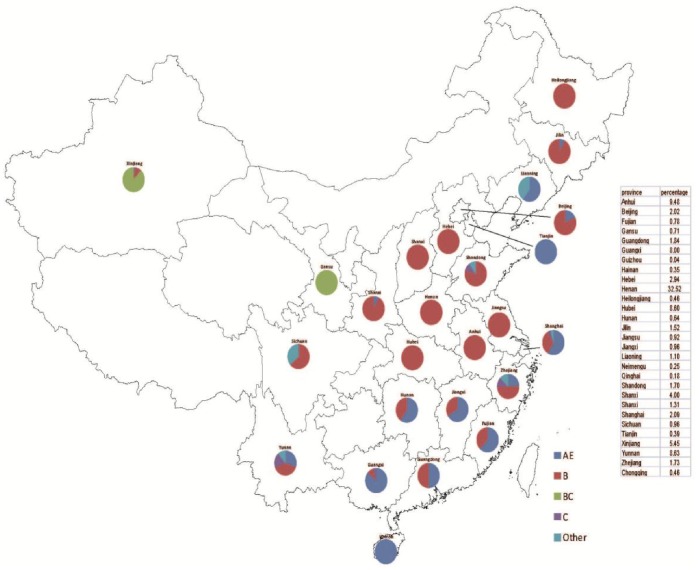
Distribution of subjects and subtype according to province.

The number of patients in each of the three cross-sectional surveys from 2004, 2005 and 2006 were 1051, 2755, and 2689, respectively. 3667 were unique individuals, with 2826 treatment-experienced and 841 treatment-naïve. Only the treatment-experienced patients were included in our analysis, whose characteristics were shown in Table 1. The patients were primarily farmers who were former plasma donors from Henan, Anhui and Hubei province. This is the cohort that has been treated the longest in China. 47.8% was male, mean age was 39.5±9.8, 85.0% was Han ethnicity, 53.9% had primary school education or less. The distribution of subjects and subtype according to province was shown in Figure 2.

**Table 1:** Factors associated with HIVDR among ART treated patients.

Variable	Number	HIV drug resistance*N* (%)	Crude OR(95% CI)	*P-* *value*	Adjusted OR (95% CI)	*P-* *value*
Total	2826	543(19.2)				
Sex						
Male	1475	274(18.6)				
Female	1351	269(19.9)	1.1(0.9,1.3)	0.37		
Age (Years)						
≤30	429	62(14.5)				
31–50	2006	395(19.7)	1.5(1.1,1.9)	0.12		
>50	391	86(22.0)	1.7(1.2,2.4)	<0.01		
Ethnicity						
Han	2403	493(20.5)				
Minorities	423	50(11.8)	0.5(0.4,0.7)	<0.01		
Married						
Yes	2097	414(19.7)				
No	729	129(17.7)	(0.9,1.7,1.1)	0.23		
Education						
Primary school or less	1524	355(23.3)				
Junior high school or more	1302	188(14.4)	0.6(0.5,0.7)	<0.01		
Farmer						
Yes	1354	313(23.1)				
No	1472	230(15.6)	0.6(0.5,0.7)	<0.01		
HIV transmission route						
Sexual intercourse	618	59(9.6)				
Blood/plasma transmission	1741	424(24.4)	3.1(2.3,4.1)	<0.01		
Drug injection	226	27(12.0)	1.3(0.8,2.1)	0.31		
Other	241	33(13.7)	1.5(0.9,2.4)	0.08		
Initial HAART Regimen						
AZT/D4T+DDI+NVP/EFV	1314	355(27.0)				
AZT/D4T+3TC+NVP/EFV	1131	105(9.3)	0.3(0.2,0.4)	<0.01	0.5(0.3,0.6)	<0.01
Other regimens	381	83(21.8)	0.8(0.6,1.0)	0.04	0.9(0.7,1.3)	0.73
Duration of HAART (months)						
0–11	996	148(14.9)				
12–23	852	176(20.7)	1.5(1.2,1.9)	<0.01	1.4(1.1,1.8)	0.01
24–35	679	157(23.1)	1.7(1.3,2.2)	<0.01	1.3(1.0,1.8)	0.05
≥36	299	62(20.7)	1.5(1.1,2.1)	0.02	1.1(0.8,1.6)	0.52
Missing doses in the past month						
No	2465	449(18.2)				
Yes	268	69(25.8)	1.6(1.2,2.1)	<0.01	1.5(1.1,2.1)	<0.01
Discontinuation	93	25(26.9)	1.7(1.0,2.6)	<0.01	0.9(0.7,1.3)	0.74
ART drug distribution location						
County hospital or CDC	1211	142(11.7)				
Township hospital or village clinic or medication monitor	1615	401(24.8)	2.5(2.1,3.1)	<0.01	1.4(1.1,1.8)	0.02
CD4 cell account at survey						
≥350	1078	133(12.3)				
200–349	862	162(18.8)	1.6(1.3,2.1)	<0.01	2.1(1.6,2.7)	<0.01
50–199	738	189(25.6)	1.4(1.9,3.1)	<0.01	3.7(2.9,4.9)	<0.01
<50	148	59(39.9)	4.7(3.2,6.9)	<0.01	5.9(3.9,8.8)	<0.01
Changing Regimens						
No	2387	458(19.2)				
Yes(3TC based Regimens)	298	54(18.1)	0.9(0.7,1.3)	0.66		
Yes(DDI based Regimens)	141	31(22.0)	1.2(0.8,1.8)	0.41		
Province						
Others	1396	145(10.4)				
Henan, Anhui and Hubei	1430	398(27.8)	3.3(2.7,4.1)	<0.01	2.2(1.7,2.9)	<0.01

### Use of HIV ART Drugs and Virologic and Immunologic Profiles

Initial HAART regimens used were AZT+DDI+NVP (24.2%), D4T+DDI+NVP (17.6%), AZT+DDI+EFV (3.1%), D4T+DDI+EFV(1.6%), AZT+3TC+NVP (13.0%), D4T+3TC+NVP (21.1%), AZT+3TC+EFV (3.0%), D4T+3TC+EFV (2.9%), and other (13.5%). DDI-based regimens were used in the earlier years but those were switched to lamivudine starting in 2005, when lamivudine became widely available [14]. At the time of their survey, the median duration of treatment was 17.4 months (interquartile range [IQR], 8.6–28.4), with 954 (33.8%) of treated patients having a VL ≥1000 copies/ml. Of these, 543 (56.9%) had resistance mutations identified, including 294 (54.1%) with dual-class resistance.

### Prevalence of HIV Drug Resistance Mutations

Among those with HIVDR mutations identified, 522/543 (96.1%) treatment-experienced patients were resistant to non-nucleoside reverse transcriptase inhibitor (NNRTI) drugs (Table 2). 311/543(57.3%) patients had HIV-1 with resistance mutations to nucleoside reverse transcriptase inhibitors (NNRTIs) 15/543 (2.8%) had major PI resistance mutations identified. All of them had received protease inhibitors. The most common NNRTI mutations occured at positions 103 and 181 in reverse transcriptase (RT) region; NRTI mutations were most common at condons 215 and 184 in RT region; and PI mutations were most frequently seen at condon 82 in protease (PR) region.

**Table 2:** HIV detectable drug limiting resistance mutations among 543 patients with plasma HIV-1 RNA concentrations ≥1000 copies/ml and drug resistance.

Mutation alleles	Number	Percentage
NNRTI (total)	522	96.1
K103H/N/S/T	287	52.9
Y181C/I/V	211	38.9
G190A/S/E/T	141	26.0
K101E/H/P	49	9.0
Y188C/H/L	29	5.3
V106A/M	21	3.9
K238T	11	2.0
F227L	14	2.6
P225H	6	1.1
M230L	2	0.4
V179F	5	0.9
L100I	1	0.2
E138K/Q	3	0.6
A98G	11	2.0
NRTI (total)	311	57.3
T215F/I/S/V/Y/	156	28.7
M184I/V	100	18.4
D67G/N	92	16.9
M41L	84	15.5
K70E/G/R	64	11.8
L210W	54	9.9
K219E/N/Q	54	9.9
K65R	29	5.3
Q151L/M	26	4.8
V75A/M/T	19	3.5
T69D/I	17	3.1
L74V/I	16	2.9
Y115F	1	0.2
PI (total)	15	2.8
V82T/A/F	4	0.7
I54IV	3	0.6
G73C	1	0.2
N88D	1	0.2
I50IV	1	0.2
I54S	1	0.2
I84IV	1	0.2
L90M	1	0.2
V32I	1	0.2
M46I	1	0.2

### Risk Factors for HIV Drug Resistance

The risk factors for HIVDR that were significantly in the univariate logistic regression analysis were considered for inclusion in the multivariate logistic regression. In the multivariate logistic regression model (Table 1), the factors significantly associated with drug resistance were province (compared to other provinces: Henan, Anhui and Hubei adjusted odds ratio [AOR] 2.2, 95% confidence interval [CI] 1.7–2.9); Duration of HAART (compared to less than 12 months: 12–23 AOR 1.4, 95% CI 1.1–1.8, 24–35 AOR 1.3, 95% CI 1.0–1.8, ≥36 AOR 1.1, 95% CI 0.8–1.6); CD4 cell count at survey (compared to CD4≥350/ul: CD4 200–349 AOR 2.1, 95% CI 1.6–2.7, CD4 50–199 AOR 3.7, 95% CI 2.9–4.9, CD4 0–49/ul AOR 5.9, 95% CI 3.9–8.8); ART drug distribution location(compared with county hospital or CDC, AOR 1.4, 95% CI 1.1–1.8) and missing doses in the past month (compared to not missing any doses: AOR 1.5, 95% CI 1.1–2.1, discontinuation: AOR 0.9, 95% CI 0.7–1.3). Compared to didanosine-based regimens, lamivudine-based regimens were protective against developing drug resistance (AOR 0.5, 95% CI 0.3–0.6).

## Discussion

This analysis of data from the Chinese National HIVDR Surveillance and Monitoring Network showed that among 2826 treatment-experienced patients, 33.8% had a viral load ≥1000 copies/mL and 19.2% had resistance mutations identified, virtually all with NNRTI mutations and two-thirds with NRTI mutations. Patients at the greatest risk of HIVDR were those who received care at township hospitals or village clinics; from Henan, Hubei, or Anhui; began with low baseline CD4 cell counts; started with a didanosine-based regimen; and missed doses in the previous month.

Of note, among the 954 patients with VL ≥1000 copies/mL and virus successfully sequenced, 411 (43.1%) had no resistance mutations identified, among whom 23 (5.6%) had stopped treatment; compared with 9 (1.7%) in 543 patients with mutations, this suggests that poor adherence continues to be a significant problem. Among those with mutations, the actual resistance mutations identified are not surprising for a developing country treatment program based on NRTIs and NNRTIs, with second-line PI regimens not yet scaled up. Of concern, among 543 treatment-experienced patients with drug resistance mutations, 54.1% harbored dual-class resistance. Other studies from low and middle-income countries have found a similar pattern [15]. M184I/V and K103N were the most prevalent NRTI and NNRTI mutations in our study. M184I/V confers resistance to lamivudine, which is also often the first mutation to develop in patients receiving partially suppressive triple combination therapy including lamivudine [16]. Of note, 12.5% (68/543) of the patients had ≥3 Thymidine analogues Mutations (TAMs), which confer to resistance to all NRTIs. K103N is one of the most clinically important NNRTI resistance mutations, causing 20- to 50-fold resistance to most available NNRTIs [17], [18], with its high frequency not surprising given the prevalent use of nevirapine in China. Fortunately, each patient with resistance to PI had a single major PI mutation, and none of patients were resistant to LPV/r, which is an important component of second line regimens.

Among treatment-experienced patients, adjusted risk factors for HIVDR included didanosine-based regimens (compared to lamivudine-based), care received at township hospital or village clinic, poor adherence and low baseline CD4 counts. Two factors unique to China were provinces, particularly Henan, Hubei, and Anhui, and plasma donors. They likely have more HIVDR than other areas because they are the center of the plasma donor HIV epidemic in the early-mid 1990s [9] and consequently the places where HIV treatment were first scaled up in China [2]. However, their increased HIVDR risk of 2.2 (95% CI 1.1–2.1) compared to other provinces is independent of treatment duration and route of transmission. A Chinese national survey had shown that treatment-naive HIV/AIDS patients had a low prevalence of primary resistance (3.8%), with no significant differences among the three high risk groups (former blood and plasma donors, sexually infected individuals, and intravenous drug users) [12]. Therefore, the higher rate of HIVDR in these provinces is not due to exposure to suboptimal therapies prior to cART treatment through the National Free Antiretroviral Treatment Program. In addition, our findings show that patients and healthcare providers from poor, rural areas, such as those from these provinces, were significantly more likely to have HIVDR than those received care in county hospital or CDC, where medical resources may be limited and staff members may have lower levels of education and less advanced technology available [19].

This analysis of HIVDR by the Chinese National HIVDR Surveillance and Monitoring Network is notable for several reasons. First, the significant proportion of patients in virologic failure but without resistance mutations identified and the lag time between time to virologic failure and time to HIVDR suggest that poor treatment adherence continues to play a major role in the development of HIVDR. Second, a significantly higher risk of HIVDR was noted among treatment-experienced patients from Henan, Hubei, and Anhui. This demonstrates that rates and patterns of HIVDR are not constant across China and that local factors play a significant role in the development of HIVDR. In-depth analyses of these provinces are needed to understand better these local factors and how to respond to them. In addition, baseline HIVDR testing should be considered before initiating treatment in patients from these provinces. Finally, this analysis underscores the need to expand access to newer antiretroviral drugs in China, including new HIV drug classes. With increasing rates of HIVDR and second-line therapy slowly scaling up, the need for future additional treatment options is clear and must be a priority in China’s efforts to control HIV/AIDS.

## References

[1] Zhang F, Dou Z, Ma Y, Zhao Y, Liu Z, et al.. (2009) Five-year outcomes of the China National Free Antiretroviral Treatment Program. Ann Intern Med 151: 241–251, W-252. 10.7326/0003-4819-151-4-200908180-00006 19687491

[2] Zhang F, Haberer JE, Wang Y, Zhao Y, Ma Y, et al (2007) The Chinese free antiretroviral treatment program: challenges and responses. AIDS 21 Suppl 8S143–148. 18172383 10.1097/01.aids.0000304710.10036.2b

[3] DeGruttola V, Dix L, D’Aquila R, Holder D, Phillips A, et al (2000) The relation between baseline HIV drug resistance and response to antiretroviral therapy: re-analysis of retrospective and prospective studies using a standardized data analysis plan. Antivir Ther 5: 41–48. 10846592 10.1177/135965350000500112

[4] D’Aquila RT, Johnson VA, Welles SL, Japour AJ, Kuritzkes DR, et al (1995) Zidovudine resistance and HIV-1 disease progression during antiretroviral therapy. AIDS Clinical Trials Group Protocol 116B/117 Team and the Virology Committee Resistance Working Group. Ann Intern Med 122: 401–408. 7856987 10.7326/0003-4819-122-6-199503150-00001

[5] Zolopa AR, Shafer RW, Warford A, Montoya JG, Hsu P, et al (1999) HIV-1 genotypic resistance patterns predict response to saquinavir-ritonavir therapy in patients in whom previous protease inhibitor therapy had failed. Ann Intern Med 131: 813–821. 10610625 10.7326/0003-4819-131-11-199912070-00003 PMC2606144

[6] Boden D, Hurley A, Zhang L, Cao Y, Guo Y, et al (1999) HIV-1 drug resistance in newly infected individuals. JAMA 282: 1135–1141. 10501116 10.1001/jama.282.12.1135

[7] Ma Y, Zhang F, Zhao Y, Zang C, Zhao D, et al (2010) Cohort profile: the Chinese national free antiretroviral treatment cohort. Int J Epidemiol 39: 973–979. 19556327 10.1093/ije/dyp233 PMC2929349

[8] Zhang F, Dou Z, Ma Y, Zhang Y, Zhao Y, et al (2011) Effect of earlier initiation of antiretroviral treatment and increased treatment coverage on HIV-related mortality in China: a national observational cohort study. Lancet Infect Dis 11: 516–524. 21600849 10.1016/S1473-3099(11)70097-4

[9] Dou Z, Chen RY, Wang Z, Ji G, Peng G, et al (2010) HIV-infected former plasma donors in rural Central China: from infection to survival outcomes, 1985–2008. PLoS One 5: e13737. 21060835 10.1371/journal.pone.0013737 PMC2966407

[10] Zhang F (2008) National Free HIV Antiretroviral Treatment Handbook. People’s Medical Publishing House.

[11] Zhong P, Pan Q, Ning Z, Xue Y, Gong J, et al (2007) Genetic diversity and drug resistance of human immunodeficiency virus type 1 (HIV-1) strains circulating in Shanghai. AIDS Res Hum Retroviruses 23: 847–856. 17678466 10.1089/aid.2006.0196

[12] Liao L, Xing H, Shang H, Li J, Zhong P, et al (2010) The prevalence of transmitted antiretroviral drug resistance in treatment-naive HIV-infected individuals in China. J Acquir Immune Defic Syndr 53 Suppl 1S10–14. 20104099 10.1097/QAI.0b013e3181c7d363 PMC3422681

[13] Liu TF, Shafer RW (2006) Web resources for HIV type 1 genotypic-resistance test interpretation. Clin Infect Dis 42: 1608–1618. 16652319 10.1086/503914 PMC2547473

[14] Dou Z, Chen RY, Xu J, Ma Y, Jiao JH, et al (2010) Changing baseline characteristics among patients in the China National Free Antiretroviral Treatment Program, 2002–09. Int J Epidemiol 39 Suppl 2ii56–64. 21113038 10.1093/ije/dyq215 PMC2992620

[15] Garrido C, Zahonero N, Fernandes D, Serrano D, Silva AR, et al (2008) Subtype variability, virological response and drug resistance assessed on dried blood spots collected from HIV patients on antiretroviral therapy in Angola. J Antimicrob Chemother 61: 694–698. 18218644 10.1093/jac/dkm515

[16] Tisdale M, Kemp SD, Parry NR, Larder BA (1993) Rapid in vitro selection of human immunodeficiency virus type 1 resistant to 3′-thiacytidine inhibitors due to a mutation in the YMDD region of reverse transcriptase. Proc Natl Acad Sci U S A 90: 5653–5656. 7685907 10.1073/pnas.90.12.5653 PMC46779

[17] Casado JL, Hertogs K, Ruiz L, Dronda F, Van Cauwenberge A, et al (2000) Non-nucleoside reverse transcriptase inhibitor resistance among patients failing a nevirapine plus protease inhibitor-containing regimen. AIDS 14: F1–7. 10708276 10.1097/00002030-200001280-00001

[18] Joly V, Moroni M, Concia E, Lazzarin A, Hirschel B, et al (2000) Delavirdine in combination with zidovudine in treatment of human immunodeficiency virus type 1-infected patients: evaluation of efficacy and emergence of viral resistance in a randomized, comparative phase III trial. The M/3331/0013B Study Group. Antimicrob Agents Chemother 44: 3155–3157. 11036040 10.1128/aac.44.11.3155-3157.2000 PMC101620

[19] Ruan Y, Xing H, Wang X, Tang H, Wang Z, et al (2010) Virologic outcomes of first-line HAART and associated factors among Chinese patients with HIV in three sentinel antiretroviral treatment sites. Trop Med Int Health 15: 1357–1363. 20868414 10.1111/j.1365-3156.2010.02621.x

